# Is It All about Endothelial Dysfunction? Focusing on the Alteration in Endothelial Integrity as a Key Determinant of Different Pathological Mechanisms

**DOI:** 10.3390/biomedicines10112757

**Published:** 2022-10-31

**Authors:** Pasquale Ambrosino, Silvestro Ennio D’Anna, Guido Grassi, Mauro Maniscalco

**Affiliations:** 1 Istituti Clinici Scientifici Maugeri IRCCS, Cardiac Rehabilitation Unit of Telese Terme Institute, 82037 Telese Terme, Italy; 2 Istituti Clinici Scientifici Maugeri IRCCS, Pulmonary Rehabilitation Unit of Telese Terme Institute, 82037 Telese Terme, Italy; 3 Clinica Medica, Department of Medical Sciences, Milano-Bicocca University, 20126 Milan, Italy; 4 Department of Clinical Medicine and Surgery, Federico II University, 80131 Naples, Italy

The endothelium is composed of a monolayer of endothelial cells (ECs) covering the inner side of arterial, venous and lymphatic vessels [1]. ECs display important homeostatic functions, since they are able to respond to both humoral and hemodynamic triggers [2]. Being present in virtually every part of the body, the endothelium plays a key role in the systemic control of vascular tone, inflammation, coagulation and oxidative stress [3,4,5]. Accordingly, endothelial dysfunction has been identified as a key and early pathogenic mechanism in many clinical conditions [6], including the novel coronavirus disease 2019 (COVID-19) and its post-acute sequelae [7,8]. The leading role of endothelial function in vascular and organ homeostasis has led to the development of many clinical and laboratory tests for its assessment [9]. Furthermore, considering its pathogenetic role, the loss of functional integrity of ECs has been proposed as a potential therapeutic target for tailored pharmacological, rehabilitation and even epigenetic approaches [10].

Numerous original [11,12,13,14,15,16,17,18] and review articles [19,20,21,22,23,24] were published in the first volume of this Special Issue, entitled “Endothelial Dysfunction: From a Pathophysiological Mechanism to a Potential Therapeutic Target” [25], contributing to an improved understanding of the intrinsic mechanisms of endothelial dysfunction and elucidating many aspects of the relationship between the alteration in endothelial integrity and the onset of inflammation, oxidative stress and hypercoagulable states. Thus, the pathogenesis of endothelial dysfunction was analyzed and discussed in light of the contributing role of the Notch pathway [19] and neutrophil extracellular traps and NLR family pyrin-domain-containing 3 (NLRP3) inflammasome [21]. Moreover, the complex interplay between ECs and the sympathetic nervous system was extensively reviewed by Quarti-Trevano et al. [22], who proposed it as a key pathogenic mechanism in heart failure and hypertension. Accordingly, in another interesting article [16], the prognostic role of endothelial dysfunction was confirmed in hypertensive subjects as a surrogate marker of cardiovascular risk and a predictor of future diabetes onset. The potential therapeutic and clinical implications of endothelial damage were also analyzed in other clinical conditions, including autoimmune, infective and multifactorial diseases. Thus, Lo Gullo et al. [12] reported a direct association between the severity of pulmonary impairment and the degree of endothelial dysfunction in patients with systemic sclerosis (SSc), as expressed by circulating endocan levels. Another functional study [15] suggested the potential beneficial effect of multidisciplinary rehabilitation on endothelium-dependent flow-mediated dilation (FMD) of convalescent COVID-19 patients referred to a post-acute care setting, thus supporting the hypothesis that exercise-based interventions may reduce endothelial dysfunction and cardiovascular risk in the post-acute phase of COVID-19 [7]. The beneficial impact of gliflozins on endothelial function, due to a reduction in oxidative stress and inflammation, was also discussed in another intriguing paper [23].

Overall, this first collection of articles offered a comprehensive but non-exhaustive view of the molecular pathways related to the homeostatic functions of the endothelium, with a focus on the clinical implications of endothelial dysfunction in different clinical settings. However, in response to the need for further preclinical and translational research on this issue, a second volume has been launched, with some clinical and laboratory studies already published.

In line with the widespread belief that endothelial dysfunction is the earliest stage of atherosclerosis, Coppola et al. [26] published an in vitro study in human EC cultures (Ea.hy926) to explore the role of vascular calcification as the leading cause of cardiovascular mortality in chronic kidney disease (CKD). In particular, the authors demonstrated that the uremic toxin lanthionine, generated as a side-product of the trans-sulphuration process, is able to modify endothelial homeostasis at a concentration similar to that usually documented in CKD patients, with the expression of specific markers involved in the mineralization process. Another laboratory study [27] used mouse-brain microvascular endothelial cells (bMECs) to explore the antioxidant, anti-inflammatory and neuroprotective effects of resveratrol, a phenolic compound of grape, mulberry and blueberry peels. First, the authors demonstrated that interleukin (IL)-1β is able to induce the expression of matrix metalloproteinase-9 (MMP-9) in bMECs, thus causing the disruption of the arranged integrity of zonula occludens-1 (ZO-1). Hence, they found that resveratrol can reduce the upregulation of MMP-9 caused by IL-1β in bMECs. Exploring the hypothesis that sodium (Na^+^) accumulation in the skin may be responsible for salt sensitivity in salt-sensitive hypertension, Šilhavý et al. [28] analyzed skin Na^+^ levels after 10 days of salt loading in salt-sensitive hypertensive rats (SHRs) and salt-resistant normotensive Brown Norway (BN-Lχ) rats. Interestingly, the authors documented significantly increased salt storage in SHR after loading. Furthermore, the BN-Lχ rats exhibited an upregulation of the genes involved in endothelial proliferation and angiogenesis, along with a significant increase in skin capillary density. The authors hypothesized that skin vasodilation is a normal response to salt loading in salt-resistant rats, along with a subsequent decrease in systemic vascular resistance. In contrast, they speculated that salt sensitivity may be associated with endothelial dysfunction and capillary rarefaction in the skin, causing a failure to vasodilate and reduce systemic vascular resistance. Two clinical studies [29,30] have also been published in this second volume. In a small cohort of patients with peripheral artery disease (PAD) [29], vascular endothelial growth factor (VEGF) levels were measured in ischemic and nonischemic skeletal muscles before and after revascularization. Significantly reduced VEGF levels were found in ischemic muscles, particularly in advanced PAD stages and diabetic patients, with a significant increase after endovascular or surgical restoration of vascular patency. These interesting findings support the hypothesis of a local endothelial compensatory mechanism against hypoxia in PAD mediated by the increase in VEGF levels and consequent angiogenesis. Among patients with stable coronary artery disease, Sbrana et al. [30] demonstrated instead the association between dense macrocalcifications in coronary plaques and the M2-like circulating monocyte fingerprint, also suggesting that trans-endothelial migration during plaque formation may be primary sustained by Mon1 (CD14^++^/CD16^−^) and Mon2 (CD14^++^/CD16^+^) circulating subsets. A comprehensive review of the complex relationship between obesity and endothelial dysfunction with a focus on the mechanisms underlying the increased cardiovascular risk of obese patients is also included in this second volume of the Special Issue [31]. In this regard, the authors summarized the literature presenting evidence for the altered function of adipocytes in obesity, which may determine increased levels of pro-inflammatory adipokines (i.e., IL-1β, IL-6, tumor necrosis factor-α, transforming growth factor-β, serum amyloid A, leptin, resistin, osteopontin); reduced levels of anti-inflammatory, antithrombotic and antidiabetic adipokines (i.e., IL-10, adiponectin, omentin, pigment-epithelium-derived factor, secreted frizzled-related protein, C1q/TNF-related proteins); and the consequent systemic enhancement of inflammation, oxidative stress and endothelial dysfunction. The article also summarized the lifestyle interventions, including exercise and diet, that may positively impact endothelial function and, therefore, cardiovascular risk, in obese subjects.

Overall, a limited number of the complex and multiple molecular aspects of endothelial homeostasis were analyzed in this relatively small collection of articles, from which it seems clear that the alteration in endothelial integrity may be a key pathological mechanism in different clinical conditions, including hypertension, heart failure, coronary artery disease, CKD, autoimmune diseases, COVID-19 and post-acute COVID-19 syndrome. One of the recurring themes is the role of endothelial dysfunction as an early event of the atherosclerotic process and plaque formation, which suggests that monitoring endothelial function through clinical (e.g., FMD) and laboratory methods may be used as a surrogate marker of cardiovascular risk. In this regard, the epidemiological evidence of an increased cardiovascular risk in any clinical condition characterized by chronic inflammation, regardless of its etiology [32,33], is consistent with the contents of some papers included in both the first and second volumes of the Special Issue. Another translational aspect that has emerged is the possibility of directly treating endothelial dysfunction, not only as a welcome pleiotropic effect of routine pharmacological and rehabilitation strategies but also as a consequence of novel approaches specific to the endothelium or at least driven by its response. In this regard, based on a better knowledge of the mechanisms underlying endothelial homeostasis, some promising candidates and epigenetic approaches have been proposed and tested, with encouraging findings [10,34].

In light of current evidence, it is reasonable to assume that many other diseases may be impacted by an imbalanced endothelial homeostasis, with a number of deregulated pathways still to be elucidated. The endothelium is ubiquitous in humans, being the largest organ of the body with complex functions, which display their specificity in different organs and tissues. Therefore, more translational and laboratory research is urgently needed for a better understanding of such homeostatic functions of the endothelium and a deeper awareness of how and to what extent the alteration in endothelial integrity can represent a key determinant of different disease mechanisms. This second volume of the Special Issue, entitled “Endothelial Dysfunction: From a Pathophysiological Mechanism to a Potential Therapeutic Target (Volume II)”, focuses on the mechanisms and diagnosis, as well as the potential prognostic and therapeutic implications of endothelial dysfunction as a biomarker of inflammation, oxidative stress and vascular disease.
